# Frequency distribution of HCV genotypes among chronic hepatitis C patients of Khyber Pakhtunkhwa

**DOI:** 10.1186/1743-422X-8-193

**Published:** 2011-04-26

**Authors:** Sajid Ali, Ijaz Ali, Sadiq Azam, Bashir Ahmad

**Affiliations:** 1 Centre for Biotechnology and Microbiology, University of Peshawar, Peshawar, Khyber Pakhtunkhwa, Pakistan; 2 Institute of Biotechnology and Genetic Engineering, Agricultural University, Peshawar, Khyber Pakhtunkhwa, Pakistan

**Keywords:** Hepatitis C Virus, Interferon, complementary DNA, Real-time PCR, Type specific PCR

## Abstract

**Background:**

Hepatitis C Virus (HCV) genotypes frequency is important for the predication of response to therapy and duration of treatment. Despite variable response rates experienced in the case of Interferon (IFN) -based therapies, there was scarcity of data on HCV genotypes frequency in Khyber Pakhtunkhwa (KPK).

**Study Design:**

A total of 200 blood samples were collected from chronic HCV patients prior to the initiation of anti-viral therapy. The study population included patients from 6 districts of KPK. Active HCV infection was confirmed in case of all the patients by real time PCR. HCV genotypes were determined in each case by Type-specific PCR.

**Results:**

The analysis revealed that out of 200 PCR positive samples; 78 (39%) were 2a, 62 (31%) were 3a, 16 (8%) were 3b, 34 (17%) were untypable while 1a, 2b and 1b were 3 (1.5%), 2 (1%) and 5 (2.5%), respectively.

**Conclusion:**

Genotype determination is not carried out prior to therapy in KPK. Although, the abundantly prevalent types (2a and 3a) of HCV in KPK are susceptible to combination therapy, yet resistance experienced in some of the chronic HCV patients may partly be attributed to the prevalence of less prevalent resistant genotypes (1a, 1b) of HCV among the population.

## Background

Chronic HCV infection frequently progresses to liver cirrhosis and is associated with an elevated risk for development of hepatocellular carcinoma [1-3]. Though symptoms may be mild for decades, 20% of persistently infected individuals may eventually develop severe liver disease including cirrhosis and liver cancer [1]. To date at least six major genotypes of HCV, each having multiple subtypes, have been identified worldwide [2]. The various HCV genotypes are important with respect to epidemiology, vaccine development and clinical management of chronic HCV infection [3]. HCV genotype has also been linked with sustained virological response [4]. Some studies have reported that patients with type 2 and type 3 HCV infections are more likely to have a sustained response to therapy than patients with type 1 HCV infections [5]. Sustained virological response to combination therapy in patients infected with HCV-2/3 and HCV-1 genotypes are 65% and 30%, respectively [6,7]. Therefore, HCV genotype should be determined prior to therapy.

HCV genotypes 1, 2 and 3 appear to have a worldwide distribution while subtypes 1a and 1b are the most common genotypes in the United States [4] and Europe [8-10]. The most abundant subtype in Japan is subtype 1b [11]. HCV subtypes 2a and 2b are relatively common in North America, Europe and Japan and subtype 2c is found commonly in northern Italy. HCV genotype 4 appears to be prevalent in North Africa and the Middle East [12,13] and genotypes 5 and 6 are found in South Africa and Hong Kong, respectively [14,15]. HCV genotypes 7, 8 and 9 have been identified only in Vietnamese patients [16] and genotypes 10 and 11 have been identified in patients from Indonesia [17]. There is disagreement about the number of genotypes into which HCV isolates should be classified and genotypes 7 through 11 should are regarded by some researchers as variants of the same group and classified as a single genotype, type 6 [18,19].

In Pakistan, few studies have been conducted in order to figure out the geographical distribution of various HCV genotypes [13,20,21]. In Khyber Pakhtunkhwa, the prevalence of various HCV genotypes is unknown. As HCV genotypes have earlier been implicated in response to therapy [4], therefore it was of prime importance to investigate the frequency distribution of HCV genotypes in Khyber Pakhtunkhwa province of Pakistan where genotype determination prior to therapy is not common practice.

## Methodology

Samples were collected from chronically infected patients with HCV. None of the patients selected for the study had started with anti-viral therapy. All the patients duly signed a proforma containing their demographics, previous history of viral infection etc. Serum was isolated and extraction of RNA was carried out with spin column based method (Roboscreen extraction kit) according to the manufacturer's instructions. HCV-RNA was amplified using reagents and equipment from the Cepheid (Cepheid, USA).

HCV genotyping was performed according to the procedure of Ohno et al., [22]. Briefly, HCV-RNA was reverse transcribed to cDNA using 100 U of M-MLV reverse transcriptase at 37°C for 50 minutes. Two µl of synthesized cDNA was used for subsequent PCR amplification of 470-bp region from HCV 5' noncoding region plus core region by first round PCR. The second round nested PCR was performed using Type-specific primers for genotypes 1a, 1b, 1c, 3a, 3c, 4, 2a, 2c, 3b, 5a and 6a. The final PCR product was electrophorased on a 2% agarose gel to separate type-specific amplified fragments. A 100-bp DNA ladder (Invitrogen, Corp., California, USA) was run in each gel as DNA size marker and the HCV genotype for each sample was determined by identifying the HCV genotype-specific PCR band.

## Results

HCV genotype analysis in chronic HCV patients (age 15-60 years) of Khyber Pakhtunkhwa revealed that the most abundant genotypes/subtypes among the patients analyzed were 2a followed by subtype 3a (Table 1). Other common genotypes included the untypable type of the virus and genotype 3b. Comparatively less common genotypes were 1b, 1a and 2b (Table 1). Major risk factors associated with HCV infection were blood transfusion and dental surgery followed by major surgery (Table 2). The patient's history indicated that comparatively more patients had a history of blood transfusion in Peshawar and Mardan districts while a history of dental surgery practices was common in less developed district Bunir. Moreover, district Bunir and Peshawar had more cases of a positive family history of HCV infection as well. In the studied individuals, there were no co-infections. It was also noted that the overall prevalence of chronic HCV was more in male as compared to female (Table 2).

**Table 1:** Prevalent HCV genotypes among the Chronic HCV infected patients of Khyber Pakhtunkhwa.

S. No	Genotypes	Subtypes	No of isolates	% Frequency	T. No of isolates
1	2	2a	78	39%	80
			
		2b	2	1%	

2	3	3a	62	31%	78
			
		3b	16	8%	

3	1	1a	3	1.5%	8
			
		1b	5	2.5%	

4	Untypable	Untypable	34	17%	34

			Total	100	200

**Table 2:** Demographics of Chronic HCV infected patients.

District	Age	M/F Ratio	Family History of HCV infection	Co-infection	Major surgery	Blood transfusion	Dental surgery
Bunir	22-56	11/5	5%	Nil	2%	5%	9%

Swabi	18-53	13/8	3%	=	4%	6%	7%

Dir	16-58	18/12	2%	=	3%	5%	8%

Kohat	20-45	16/9	2%	=	3%	4%	6%

Mardan	19-56	29/18	3%	=	4%	8%	7%

Peshawar	15-60	41/23	4%	=	4%	9%	5%

## Discussion

HCV prevalence is alarmingly high in Pakistan and due to unsatisfactory health care conditions, the infection is spreading at a rapid pace [20]. In Khyber Pakhtunkhwa, where the health care facilities are poorly equipped with essentials for screening and sterilization, HCV has become an economic burden over a population with considerable number of people living below the poverty line.

HCV genotype determination facilitates therapeutic decisions and strategies [23,24] and it has been reported that pathogenesis of the disease or response to therapy may vary according to the type of the virus [9,25]. Genotype determination is not carried out prior to treatment in Khyber Pakhtunkhwa and the treatment strategies are solely based on qualitative or quantitative viral detection. Reasons for variable response rates in the case of different patients for both combination therapy and Pegylated IFN could therefore not properly be sorted out. We attempted to figure out HCV genotypes prevalent among the population of Khyber Pakhtunkhwa so as to create awareness among the practitioners about the importance of the HCV genotype and find a preliminary answer to the resistance phenomenon experienced here.

Our study indicated that HCV genotype 2a is the most abundant followed by 3a and 2b (Table 1). The distribution of type 2 and 3 is thought to be worldwide [4] including Pakistan [10]. It has earlier been reported that HCV 2a and 3a are most susceptible to combination therapy [5] and good response rates in Khyber Pakhtunkhwa may partly be attributed to the most abundant types of HCV prevalent here. Other types of the virus including type 1a, 1b and 2b are less prevalent among the population (Table 1). Genotype 1a and 1b are prevalent in the USA [4], Europe [8,9] and Japan [11]. These genotypes are comparatively less responsive to combination therapy [5] and the response rates in the case of Pegylated IFN have been reported to vary from 50-70% [26]. In Khyber Pakhtunkhwa, it is a common practice to put the non-responders on prolonged combination therapy or Pegasys. Resistance in some patients may be attributed to these less prevalent genotypes among the population.

HCV genotypes among a considerable number of chronic HCV patients (17%) could not be determined by the assay used in this study. Untypeable genotypes have earlier been reported by other studies conducted in Pakistan [23]. Sequencing of the untypeable genotypes is needed not only for academic reasons but also for future treatment directions.

This study concludes that the most abundant types of HCV among chronic HCV patients of Khyber Pakhtunkhwa province of Pakistan are HCV type 2a, 3a and 3b. Although these abundant types are responsive to combination therapy and Pegasys, yet proper diagnostic and treatment strategies should be adopted in order to minimize the economic burden on the population and to prevent people from an extra stress of undergoing successive therapies.

## Competing interests

The authors declare that they have no competing interests.

## Authors' contributions

SA (research scholar) carried out sampling and experimental work, BA supervised the research work conducted by SA and designed the experimental work and manuscript preparation with the help of IA (Co-supervisor). SA helped in manuscript reviewing and corrections prepared by research scholar. The final manuscript is approved by all of the authors after reviewing it critically.
